# Exploring the Driving Factors of Remote Sensing Ecological Index Changes from the Perspective of Geospatial Differentiation: A Case Study of the Weihe River Basin, China

**DOI:** 10.3390/ijerph191710930

**Published:** 2022-09-01

**Authors:** Kaili Zhang, Rongrong Feng, Zhicheng Zhang, Chun Deng, Hongjuan Zhang, Kang Liu

**Affiliations:** 1 College of Urban and Environmental Sciences, Northwest University, Xi’an 710127, China; 2 Key Research Institute of Yellow River Civilization and Sustainable Development & Collaborative Innovation Center on Yellow River Civilization Jointly Built by Henan Province and Ministry of Education, Henan University, Kaifeng 475001, China; 3 National Forestry and Grassland Administration Urban Forest Ecosystem Research Station, Xi’an 710127, China

**Keywords:** Google Earth Engine, remote sensing ecological index, eco-environment quality, multiscale geographically weighted regression model (MGWR), geographic detector

## Abstract

Using the Google Earth Engine (GEE) platform, Moderate-resolution image spectroradiometer (MODIS) data of the Weihe River Basin from 2001 to 2021 were acquired, four ecological indicators, namely, greenness, wetness, heat, and dryness, were extracted, and the remote sensing ecological index (RSEI) was constructed through principal component analysis. In addition, the geographic detectors and a multi-scale geographic weighted regression model (MGWR) were used to identify the main driving factors of RSEI changes and capture the differences in spatial changes from different perspectives using multiple indicators. The results show that (1) the quality of the eco-environment in the Weihe River basin improved as a whole from 2001 to 2021, and the RSEI increased from 0.376 to 0.414. In terms of the RSEI grade, the medium RSEI and high RSEI areas increased significantly and the growth rate increased significantly, reaching 26.42% and 27.70%, respectively. (2) Spatially, the quality of the eco-environment in the Weihe River Basin exhibited a spatial distribution pattern that was high in the south and low in the north, among which the quality of the eco-environment in the Weihe River Basin in northern Shaanxi and northwestern Ningxia and Gansu was relatively low. In addition, during the study period, the RSEI of the Qinling Mountains in the southern part of the Weihe River Basin and the Jinghe River and Luohe River areas improved significantly. The urban area on the Guanzhong Plain in the Weihe River Basin experienced rapid economic growth, and urban expansion led to a significant decrease in the quality of the eco-environment. (3) The eco-environment quality in the Weihe River Basin is the result of the interaction of natural, anthropogenic, and landscape pattern factors. All of the interactions between the influencing factors had a stronger influence than those of the individual factors. There were significant differences between the individual drivers and the spatial variation in RSEI, suggesting that different factors dominate the variation in RSEI in different regions, and zonal management is crucial to achieving sustainable management of RSEI. The study shows that to improve the eco-environment quality of the Weihe River Basin, it is necessary to further strengthen ecological protection projects, reasonably allocate landscape elements, and strengthen the resistance and resilience of the ecosystem.

## 1. Introduction

The eco-environment is defined as “the total quantity and quality of water, land, biological and climatic resources that affect human survival and development”, and is a social–economic–natural complex system [1,2,3]. It not only provides natural resources, space, and basic material conditions for human survival, but also serves as a basic guarantee for human survival and social development. Its quality can effectively reflect the degree of harmony between human production activities and the regional environment [4]. In recent decades, the eco-environment in China has been increasingly disturbed by global climate change and human activities. For example, with the acceleration of urbanization, the discharge of wastewater, solid waste, and air pollution has increased, and environmental problems such as the urban heat island effect caused by the expansion of construction land and reduction in farmland have appeared [5,6,7,8]. Based on this, scientific evaluation of the quality of the ecological environment is essential for sustainable regional development.

Generally, the quality of the eco-environment is measured according to the actual development needs of people [9]. The current methods for evaluating the quality of the eco-environment mainly include the pressure-state-response (PSR) model [10,11,12,13,14] and the ecological index (EI) [15,16], which require measuring the biological, plant, water, land, and environmental pollution levels. Both evaluation systems require a large number of indicators, and there are still some challenges in the acquisition and construction of indicators [17]. The results of these two evaluation methods are numerical values, which cannot reflect the environmental conditions of all of the distribution surfaces and lack visualization.

In recent years, with the continuous development of the 3S (Remote Sensing, Global Position System, and Geographic Information System) technology, remote sensing has been widely used in the monitoring and evaluation of the quality of the eco-environment due to its advantages of wide data sources, large data volumes, and real-time monitoring, compared with the traditional construction of indicator systems to assess the quality of the eco-environment [18]. The normalized difference vegetation index (NDVI) is the most widely used single indicator for the assessment of the quality of the eco-environment, and it has been adopted in studies of different ecosystems [19,20,21]. The leaf area index (LAI) is another vegetation index that is commonly used to evaluate the interaction mechanism between vegetation and the eco-environment [22,23]. The enhanced vegetation index (EVI) is often used to reflect the changes in vegetation growth in an ecological context [24]. The land surface temperature (LST) is used to assess the urban heat island effect [25,26,27,28]. The drought index (RDI) and standardized precipitation index (SPI) are used to assess the drought intensity [29,30]. However, the eco-environment quality is controlled by a combination of influencing factors; so, a single ecological evaluation index cannot objectively and comprehensively reflect its ecological changes. Based on this, Xu et al. [31] proposed the construction of a remote sensing ecological Index (RSEI) based on four indicators of greenness, wetness, dryness, and heat, which relies on remote sensing technology to capture ecological index information about the evaluation area, overcoming the limitations of the PSR model and the EI index [32]. At present, the RSEI has been widely used in the assessment of the quality of the regional eco-environment. Fan et al. [33] comprehensively assessed the quality of the eco-environment in the eastern coastal areas of China by constructing RSEIs. Other researchers have also evaluated RSEI remote sensing indices in different regions such as typical basins, comprehensive land consolidation areas, and grasslands [34,35,36].

However, if the RSEI is applied on a large regional scale, the huge amount of data causes difficulties, as well as complicated data preprocessing and index calculations [37]. As a remote sensing computing platform for large-scale eco-environment quality evaluation and monitoring, the Google Earth Engine (GEE) platform can better improve the problems of missing remote sensing data, chromatic aberrations, and temporal inconsistencies [38,39]. Moreover, processing steps such as image de-clouding and mosaicking, indicator calculations and statistics, and dynamic change trend analysis can be quickly implemented [40], and users can directly perform them using the platform and specific codes.

Based on a scientific assessment of the quality of the regional ecological environment, it is vital to explore the driving mechanisms affecting the quality of the ecological environment for regional ecological conservation. In recent years, researchers have explored effective ways to improve the quality of the eco-environment by studying the influencing factors of the eco-environment. The Environmental Kuznets Curve Hypothesis (EKC) [41] proposed that the impact of economic development on environmental pollution has an inverted “U”-shaped relationship that increases first and then decreases; Liu [42] et al. (2022) explored the impact of digital finance on the synergy between economic development and eco-environment through a coupled coordination model and a spatial econometric model; Effiong (2018) et al. [43] investigated the effect of urbanization on environmental pollution in Africa through semi-parametric panel fixed-effect regression techniques; Li et al. (2021) [44] used GWR to explore the influence of natural factors on the spatial distribution of soil pollution; Wang et al. [45] (2021) used principal component analysis to explore the influence of human and natural factors on the remote sensing ecological index; in addition, Zhang et al. (2022) [46] investigated the influence of four factors of internal remote sensing ecological index (greenness, humidity, heat, and dryness) on the eco-environment quality of the upper reaches of the Yangtze River through the geographic detector model. The general influencing factors of eco-environment quality mainly include: natural factors and human factors. However, landscape pattern is considered an important indicator of landscape heterogeneity and its impact on multiple ecological processes [47,48]. In addition, there may be interactions among different influencing factors, which may have additional impacts on the eco-environment, and the geographic detection model can realize the analysis of multi-factor interaction responses. Moreover, the response of each driving factor to the quality of the regional eco-environment has spatial differences, and the multi-scale geographically weighted regression model (MGWR) can overcome the limitations of the traditional regression model, and the geographic location of the regression unit can be embedded in the regression parameters, when the model is constructed [49,50,51]. Therefore, this paper adopts geographic detectors and an MGWR to identify the dominant factors affecting regional ecological remote sensing index changes and capture differences in spatial changes, in order to provide an accurate and comprehensive explanation of changes in complex eco-environment quality.

The Weihe River Basin is the largest tributary of the Yellow River Basin in China. It not only provides a huge amount of water but also serves as an important barrier for soil and water conservation and wind and sand control, and it has an inherently fragile and sensitive eco-environment [52,53]. In addition, as the core area of China’s Guanzhong–Tianshui Economic Zone and Guanzhong Plain City Cluster, the Weihe River Basin plays a pivotal role in the social and economic development of northwestern China and has important strategic value at the regional and national levels [54,55]. However, urbanization and industrialization have increased the pressure on the eco-environment quality in the Weihe River Basin. In addition, soil erosion, serious water pollution, and ecological degradation limit the sustainable development of the study area [56,57,58,59]. The combined influence of multiple factors has accelerated the urgent need to improve the eco-environment quality in the Weihe River Basin. Therefore, scientific evaluation of ecological environment quality and identification of key influencing factors and their spatial differentiation characteristics are important links to ensure the effectiveness of ecological environment management.

In summary, in order to evaluate the temporal and spatial pattern and evolution trend of the eco-environment quality of the Weihe River Basin in a long-term series, and to explore effective ways to improve the eco-environment quality of the Weihe River Basin, this study evaluated the eco-environment quality based on the remote sensing technology indicators RSEI and GEE platforms. In addition, geo-detection and multi-scale geographic weighted regression models are used to examine the correlations between the natural and anthropogenic factors and the evolutionary process of the quality of the watershed ecosystem, so as to clarify the dominant factors affecting the eco-environment quality of the river basin, and, at the same time, reveal the influence of each dominant factor on the regional remote sensing ecological index changes. This paper provides a convenient method to capture the relationship between RSEI and driving factors from a geospatial perspective, and provides a reference for sustainable management of watershed eco-environment quality.

## 2. Materials and Methods

### 2.1. Study Area

**Figure 1:**
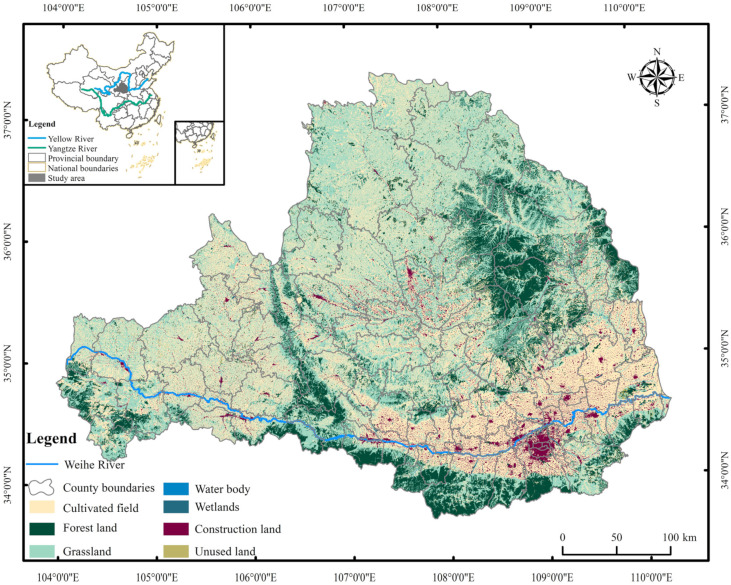
Geographic map of the Weihe River Basin of China.

The Weihe River Basin is the first sub-basin of the Yellow River Basin, in northwestern China (104°00′–110°20′ E, 33°50′–37°18′ N), with a basin area of about 1.34 × 10^5^ km^2^. It contains 13 cities in Shaanxi, Gansu, and Ningxia provinces (Figure 1). The Weihe River Basin has a continental monsoon climate zone, which is a transition zone between arid and humid areas. The precipitation in the basin decreases from southeast to northwest due to the topography. There is abundant precipitation in the south at the foot of the Qinling Mountains, with a maximum annual precipitation of 1000 mm. The precipitation decreases as the elevation of the terrain decreases, with an average annual precipitation of about 500 mm in the plain areas. The average annual temperature is 7.8–13.5 °C. The topography of the Weihe River Basin is high in the west and low in the east. The highest elevation in the west is 3495 m. From west to east, the terrain gradually becomes gentler and the valley becomes wider. The main mountain ranges are Liupanshan, Longshan, Ziwuling, and Huanglong Mountain in the north, Qinling Mountains in the south, and Taibai Mountain, the highest peak, with an altitude of 3767 m. The watershed is rich in vegetation types, mainly including coniferous forests, broad-leaved forests, scrub, grassland, grasses, and cultivated plants. The natural conditions in the basin vary significantly, and the topography is complex. The upper and middle reaches of the Wei River are the loess hill area, which is fragmented and complex, with many ravines and loose soils. This area is highly susceptible to erosion. The northern part of the middle and lower reaches of the Wei River is the Loess Plateau in northern Shaanxi, which is the area in China, and even in the world, which is the most susceptible to soil erosion. The central part of the middle and lower reaches of the Wei River is a plain area with flat terrain and an advantageous location. The southern part includes the Qinling Mountains, with high vegetation cover and good ecological conditions. The Weihe River Basin is an important part of the hydrological system in the middle and upper reaches of the Yellow River in China, and it plays an irreplaceable role in the supply of water resources and maintaining the balance of water and sediment. Under the new situation, with the development of the Guantian Economic Belt in the basin and the establishment of the Guanzhong urban agglomeration, it has brought opportunities for the economic and social development of the Weihe River Basin, and also raised challenges for the ecological protection of the basin. Therefore, scientific assessment of the eco-environment quality of the Weihe River Basin and its impact mechanism has important strategic significance for the high-quality development of the basin.

### 2.2. Data Sources

Moderate-resolution image spectroradiometer (MODIS) data are widely used in large-scale ecological research because of their advantages of a wide coverage area and short monitoring period. All of the indicators of the RSEI in this study were constructed from the MODIS remote sensing products for 2000–2021 (https://developers.google.com/earth-engine/datasets/catalog/modis (accessed on 15 April 2022)). The source and index descriptions of the four ecological indicators of greenness (normalized difference vegetation index, NDVI), wetness (WET), dryness (normalized difference building soil index, NDSI), and heat (land surface temperature, LST) are shown in Table 1. In this study, the processing of the MODIS data, including de-clouding, water body masking, cropping, and calculations, was completed using the GEE planetary cloud computing platform.

In order to explore the external driving factors affecting RSEI, we carried out geographic detection analysis from 12 indicators in three dimensions: nature, landscape, and human. The specific data sources and processing are shown in Table 2. In addition, in order to ensure the consistency of the spatial accuracy of the data, each driver was processed using the cropping and resampling tools in ArcGIS 10.5 and was converted into a uniform 1000 m × 1000 m precision raster file for geodetector analysis.

### 2.3. Research Methodology

#### 2.3.1. Remote Sensing Ecological Index

The RSEI was used in this study to monitor the ecological changes in the Weihe River Basin during 2000–2021. The four indicators of greenness (normalized difference vegetation index, NDVI), wetness (WET), dryness (normalized difference building soil index, NDSI), and heat (land surface temperature, LST) all affect human life; so, it is credible to use the coupled comprehensive index (RSEI) to reflect the changes in the quality of the eco-environment [63].

RSEI = f(NDVI, WET, NDSI, LAT)(1)

where NDVI denotes the greenness index, WET denotes the wetness index, NDSI denotes the dryness index, LST denotes the heat index, and f indicates principal component analysis (PCA).

(1) Greenness: normalized difference vegetation index

The NDVI is closely related to the vegetation growth status and biomass, which can reflect the flourishing condition of the vegetation and the quality of the regional eco-environment to some extent [64]. This NDVI is calculated as follows:

NDVI = (b_nir1_ − b_red_)/(b_nir1_ + b_red_) (2)

where b_red_ and b_nir1_ are the reflectance values of the red and near-infrared (NIR1) bands of the MOD09A1 images, respectively.

(2) Wetness: wetness component

The wetness index used in this study was the humidity component in the tasseled cap transformation, which can describe the surface environmental conditions well [65]. The calculation method is different for different data sources. The calculation formula for WET is as follows:

WET = 0.1147 × b_red_ + 0.2489 × b_nir1_ + 0.2408 × b_blue_ + 0.3132 × b_green_ −0.3122 × b_nir2_ − 0.0.6416 × b_swir1_ − 0.5087 × b_swir2_ (3)

where b_red_, b_nir1_, b_blue_, b_green_, b_nir2_, b_swir1_, and b_swir2_ are the reflectance values of the red band, NIR1 band, blue band, green band, NIR2 band, short wave infrared (SWIR1) band, and SWIR2 band in the MOD09A1 images, respectively. We determined the constant term coefficient for each band based on the evaluation of the remote sensing ecological index of China’s coastal areas by Zheng et al. (2020) [7] and the evaluation of the remote sensing ecological index of China’s Dongting Lake by Yuan et al. (2021) [32].

(3) Dryness: normalized difference building soil index

With the rapid expansion of cities, the drying of the urban area has a negative impact on the quality of the eco-environment in the area. In this study, the bare soil index (soil index, SI) and the built-up soil index (IBI) were used to construct the dryness index [66]. The NDSI was calculated as follows:

NDSI = (IBI + SI)/2(4)

IBI = {2b_swir1_/(b_swir1_ + b_nir1_) − [(b_nir1_/(b_nir1_ + b_red_)) + (b_green_/(b_green_ + b_swir1_))]}/{2b_swir1_/(b_swir1_ + b_nir1_) + [(b_nir1_/(b_nir1_ + b_red_))+ (b_green_/(b_green_ + b_swir1_))]} (5)

SI = [(b_red_ + b_swir1_) − (b_blue_ − b_nir1_)]/[(b_red_ − b_swir1_)+ (b_blue_ − b_nir1_)] (6)

where IBI is the building index; SI is the bare earth index; and b_red_, b_nir1_, b_blue_, b_green_, and b_swir1_ are the reflectance values of the red, NIR1, blue, green, and SWIR1 bands of the MOD09A1 images, respectively.

(4) Heat: land surface temperature

The GEE platform was used in this study to convert the daytime surface temperature data into actual surface temperatures. The land surface temperature (LST) was chosen as the representative of the heat index, taken from the MOD11A2 dataset, and the grayscale values were converted to the Warsaw temperature [15]. The LST was calculated as follows:

LST = 0.02 × DN − 273.15(7)

where DN is the grayscale value of the surface temperature.

(5) Water body masking

In order to avoid the influence of water bodies in the study area on the results, the Modified Normalized Difference Water Index (MNDWI) was used to mask the NDVI, WET, NDSI, and LST [67], and only the eco-environment quality of the non-water area was studied in this paper. The MNDWI was calculated as follows:

MNDWI = (b_green_ − b_swir1_)/(b_green_ + b_swir1_) (8)

where b_green_ and b_swir1_ are the reflectance values of the green band and the SWIR1 band of the MOD09A1 images, respectively.

#### 2.3.2. Principal Component Analysis

The PCA was used to concentrate the information related to the quality of the eco-environment into fewer principal components. The weight of the integrated ecological environment index does not need to be set manually; so, deviation of the weight due to subjective influence can be avoided.

The normalized greenness, humidity, heat, and dryness indicators were subjected to PCA, and the representative first principal component PC1 was extracted as the initial remote sensing ecological index RSEI′. Then, the initial RSEI was normalized to obtain the RSEI, as follows:

RSEI′ = PC1[f(NDVI, WET, NDSI, LST)](9)

(10) $$RSEI=(RSEI\prime -{RSEI}_{min}^{\prime })/({RSEI}_{max}^{\prime }-{RSEI}_{min}^{\prime })$$

where RSEI′ is the initial RSEI; ^${RSEI}_{max}^{\prime }$^ and ^${RSEI}_{min}^{\prime }$^ are the maximum and minimum values of the initial RSEI, respectively; and RSEI is the normalized RSEI (values of 0–1). The closer the RSEI is to 1, the better the quality of the regional eco-environment, and vice versa.

#### 2.3.3. Average Correlation Analysis

In order to further test the comprehensive representativeness of the RSEI, in this study, the correlation coefficients between the five indicators (RSEI, WET, NDVI, NDSI, and LST) during the study period were calculated, and the average correlation model was used to test the applicabilities of the RSEI indicators. The closer the average correlation result is to 1, the stronger the comprehensive representation of the index [1], which is expressed as follows:

S_p_ = (|S_q_| + |S_r_| + …|S_s_|)/(n − 1) (11)

where S_p_ is the average correlation; p, q, r, and s are correlation analysis indicators; n is the number of indicators in the correlation analysis; and S_q_, S_r_, and S_s_ are the Pearson correlation coefficients between the indicators.

#### 2.3.4. Geographical Detectors

Geographic probes are a powerful tool for exploratory analysis of spatial data and are a statistical method for analyzing the driving forces of various phenomena [68]. Geodetectors can not only detect and reveal factors but can also analyze the interactions between variables, and thus, they have been widely used in many fields, including ecology, environment, and social sciences. In this study, factor detection and interaction detection were used to analyze the dominant factors affecting the RSEI.

(1) Factor detector

In order to investigate the dominant factors influencing the difference in the spatial distribution of the RSEI in the study area, a factor detector was used in this study to reveal the spatial heterogeneity of the contribution of each influencing factor to the RSEI. The factor detector was calculated as follows:

(12) $$q=1-(\sum_{h=1}^{L}N_{h}\;\partial_{h}^{2}/N\partial^{2})\times \partial_{h}^{2}$$

where q is the degree of influence of the influencing factor on the dependent variable with the values of [0,1]; h = 1, 2, 3, …; L is the stratification of the influencing factor or dependent variable; N_h_ and N are the number of cells in stratum h and the entire area, respectively; and $\delta_{h}^{2}$ and δ^2^ are the variance of the dependent variable in stratum h and the entire area, respectively. The larger the value of q, the stronger its explanatory power on the dependent variable.

(2) Interaction detector

In this study, the interaction factor detector was used to explore the explanatory power of the spatial differentiation of the RSEI in the watershed under the joint influence of two different influencing factors. First, the q-value of the effect of a single factor on the dependent variable was calculated, and then, the q-value after the interaction between the two factors was calculated, and by comparing them, it was determined whether their effects on the dependent variable were enhanced or weakened.

#### 2.3.5. Global Spatial Autocorrelation

The global Moran’s I index was used to examine the spatial correlation of RSEI in the Weihe River basin, which can visualize the spatial aggregation and anomalies of RSEI. The calculation equation is as follows:

(13) $$I=(\sum_{i=1}^{n}w_{ij}(x_{i}-\bar{x})(x_{j}-\bar{x}))/(S^{2}\sum_{i=1}^{n}\sum_{j=1}^{n}w_{ij})$$

In the formula, I is the global Moran index, and the value is between (−1, 1). If I is greater than 0, it means a positive correlation; that is, the high value is close to the high value; if I is less than 0, it means a negative correlation, and the high value and the low value are close to each other. If I is close to 0, this indicates that the spatial distribution is random and there is no spatial autocorrelation; x_i_ and x_j_ are attribute values, W_ij_ is the spatial weight matrix, and S^2^ sample variance.

#### 2.3.6. Ordinary Least Squares (OLS)

The OLS model is the basic model of spatial modeling and the benchmark of analysis. OLS is a global model, and the coefficients of constants and explanatory variables in the model are the same in different study areas, and cannot reflect the spatial differences between regions. In order to ensure the accuracy of the GWR model, OLS linear regression is usually required to realize model variable diagnosis before constructing the GWR model [69]. The formula is as follows:

Y_i_ = β_0_ +β_i_X + ε_i_ (14)

where β_0_ is a constant term; β_1_ is the regression coefficient; and ε_i_ is the random error term.

#### 2.3.7. Multiscale Geographically Weighted Regression

The geographically weighted regression model (GWR) is an improved model based on the linear regression model, adding parameters reflecting geographical differences, and performing differential regression on variables in a local range, effectively avoiding errors caused by spatial differences in variables [49]. Its expression is as follows:

(15) $$Y_{i}=\beta_{0}(\mu_{i},\nu_{i})\;+\sum_{k=1}^{p}\beta_{k}(\mu_{i},\nu_{i})X_{\mathrm{ik}}+\varepsilon_{i}$$

where Y is the dependent variable (RSEI); (u_i_,v_i_) is the spatial location of the i-th sample; β_0_(u_i_,v_i_) is the intercept; p is the number of drivers; X_ik_ is the independent variable (driver); β_k_(u_i_,v_i_) represents the regression coefficient of the i-th sample on the k-th driver; ε_i_ is the error term.

The multi-scale geographically weighted regression (MGWR) model is based on the classical geographically weighted regression (GWR) model with improved bandwidth selection flaws, allowing different variables to choose different bandwidth values, better reflecting the characteristics of spatial heterogeneity among variables, and improving the accuracy of regression analysis [51]. MGWR model expressions such as formulas:

(16) $$Y_{i}=\beta_{0}(\mu_{i},\nu_{i})+\sum_{i=1}^{p}\beta_{\mathrm{bwk}}(\mu_{i},\nu_{i})X_{\mathrm{ik}}+\varepsilon_{i}$$

where bwk in β_bwk_ represents the bandwidth used to calibrate the k-th conditional relationship. In this paper, MGWR uses a Gaussian kernel function and is calibrated using the golden section search bandwidth selection routine [50]. Model calibrations were all performed by MGWR 2.0 software, and Li and Fotheringham (2020) [50] provided more detailed information about the MGWR modeling process.

## 3. Results

### 3.1. RSEI Model Test

As can be seen from Table 3, the contribution rates of the first principal component eigenvalues of the four indicators were above 60% during the study period, indicating that the first principal component maximized the concentration of the characteristics of the four indicators, namely, greenness, wetness, dryness, and heat, and the contribution rates of each indicator only had the same positive and negative distributions in the first principal component. Specifically, the NDVI, which represents vegetation cover, and Wet, which represents the wetness of the eco-environment, both had positive values in the first principal component, while the LST, which represents the surface temperature, and the NDSI, which represents the degree of hardening of the land used for construction and bare soil, both had negative values. This indicates that NDVI and Wet promote the improvement of the eco-environment quality, while the LST and NDSI inhibit the improvement of the eco-environment quality, which is consistent with the realistic influences of the four indicators on the eco-environment. Therefore, it is feasible to select the results of the first principal component for use in the RSEI calculation.

**Figure 2:**
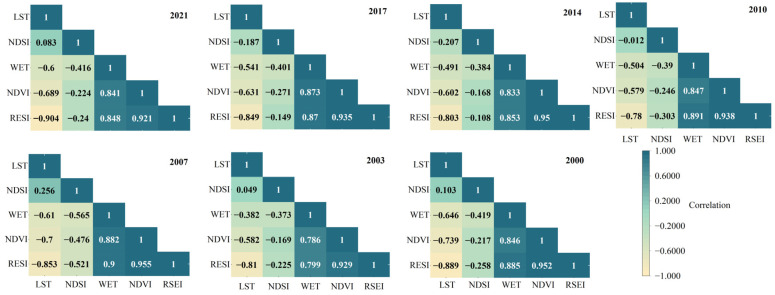
Correlation of each ecological factor with RSEI.

The Pearson correlation coefficients between the RSEI and the various ecological factors in the same period were also calculated (Figure 2), and the applicability and feasibility of the RSEI were tested based on the average correlation (Table 4). The results revealed that the correlation coefficients between each ecological factor and the RSEI during the study period were all significant at the 1% level. The RSEI was positively correlated with the WET and NDVI and negatively correlated with the NDSI and LST. The average degree of correlation of each index was as follows: RSEI > NDVI > NDSI > LST > WET. The average degree of correlation of the RSEI was the largest (0.68–0.81). The average correlations of the RSEI during the study period were 19.93%, 17.11%, 63.15%, and 39.67% higher than those of the NDVI, WET, NDSI, and LST, respectively. This further confirms the feasibility of using the RSEI model in this study and demonstrates that it is more representative than any single indicator.

### 3.2. Spatial and Temporal Patterns of Eco-Environment Quality in the Weihe River Basin

#### 3.2.1. Overall Eco-Environment Quality Trends in the Weihe River Basin

**Figure 3:**
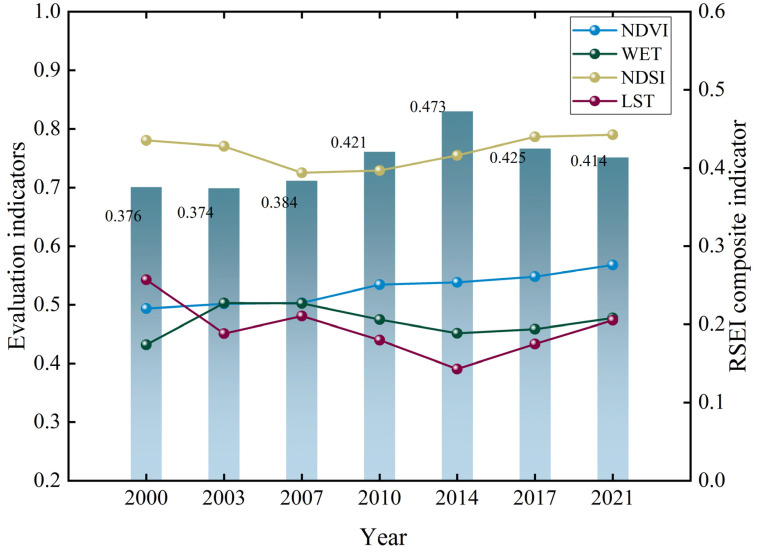
Distribution of the mean values of the single and RSEI composite indicators of the eco-environment quality in the Weihe River Basin.

Figure 3 shows the mean values and distribution of the RSEI for 7 years during 2000–2021. Figure 3 shows that the overall eco-environment quality in the Weihe River Basin improved during 2000–2021, and the mean RSEI value increased from 0.376 in 1990 to 0.414 in 2019. During 2000–2021, the mean values of the NDVI and WET, which played a positive role in the environment, exhibited a fluctuating increasing trend, while the mean values of the NDSI and LST indicators, which played a negative role, gradually decreased from 2001 to 2014, and increased in 2014. This is consistent with the trend of the RSEI composite indicators. Based on the results of the indicators; although, the negative effects of the heat factor and dryness factor increased from 2014 to 2021, under the combined effect with other influencing factors, the eco-environment was not adversely affected compared to 2001–2014, and the quality of the eco-environment improved during this period.

#### 3.2.2. Spatial Differentiation of Eco-Environment Quality in the Weihe River Basin

**Figure 4:**
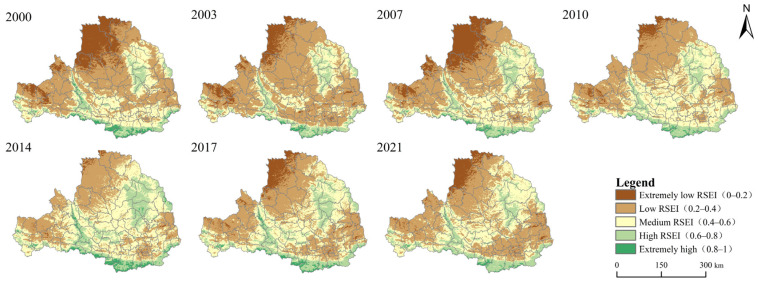
Spatial pattern of the eco-environment quality in the Weihe River Basin during 2000–2021.

Figure 4 shows the spatial distribution of the grid-scale RSEI in the Weihe River Basin. The overall ecological status of the Weihe River Basin exhibits a geographical distribution pattern of high in the south and low in the north, with significant differences in the spatial distribution. From Figure 4, it can be seen that the units in which the RSEI increased (i.e., improvement of the eco-environment quality) were mainly distributed in the central and northern parts of the Weihe River Basin. The southern part of the Weihe River Basin consists of the northern slope of the Qinling Mountains. The vegetation coverage in this area, especially the forest resources, is relatively rich, the intensity of human activities is relatively weak, and the quality of the eco-environment is relatively high. In addition, the quality of the eco-environment along the Jinghe River and Luohe River, the two major tributaries of the Weihe River Basin, also gradually improved, and this improvement gradually spread to the surrounding areas in the middle and upper levels. However, the quality of the eco-environment in the urban areas along the Weihe River exhibited a significant decrease. In addition, in northern Shaanxi and in northwestern Ningxia and Gansu, the Weihe River is located on the Loess Plateau where the overall vegetation coverage is relatively low, the soil erosion is serious, and the ecological flow is low.

#### 3.2.3. Evolution of Eco-Environment Quality Classes in the Weihe River Basin

In order to better reveal the changes in the RSEI over the 21-year study period, the RSEI values of the Weihe River Basin were divided into five classes at equal intervals of 0.2 to represent five different levels of ecological conditions (Table 5). The results show that the overall level of the quality of the eco-environment in the Weihe River Basin is relatively low, and the percentage of extremely low and low RSEI areas being greater than 50% makes ecological protection in the Weihe River Basin challenging. The proportion of areas with extremely low ecological conditions decreased by 59.13% (12.86 × 10^3^ km^2^) during the study period. The proportion of areas with low ecological conditions increased by 1.7 × 10^3^ km^2^ (2.80%). The proportion of medium and high ecological condition areas increased significantly, by 26.42% and 27.70%, respectively. Due to the fragile ecological background of the Weihe River Basin, the percentage of areas with very high ecological status was only 0.88–3.08%. During the 21-year study period, the transformation of the RSEI has mainly focused on three levels: extremely low, medium, and high RSEI areas. The proportion of extremely poor and lower areas decreased from 61.56% to 53.22%. The areas with medium RSEI values gradually increased, but the overall area ratio structure did not change significantly, which indicates that the ecological and environmental conditions in the Weihe River Basin improved during the study period, but a great deal of work is still needed to protect the eco-environment and to develop the regional economy and society in a coordinated manner in the future.

#### 3.2.4. Trends in Eco-Environment Quality Changes in the Weihe River Basin

**Figure 5:**
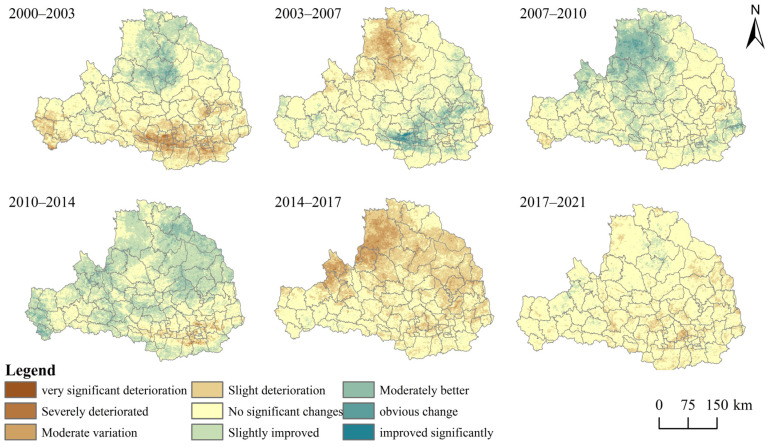
Spatial variations in the RSEI classes in the Weihe River Basin during 2000–2021.

In order to quantitatively study the temporal and spatial distribution characteristics and the trend of the changes in the quality of the eco-environment in the Weihe River Basin, the difference method was used to compare the RSEI during the seven periods and to divide them into nine categories (Figure 5). Figure 5 shows the spatial and temporal transformation levels of the RSEI for the six intervals from 2000 to 2003, 2003 to 2007, 2007 to 2010, 2010 to 2014, 2014 to 2017, and 2017 to 2021, respectively. It can be seen from Table 5 that the area in which the quality of the eco-environment remained stable accounted for a large proportion (37.65–87.34%). The proportion of the area of the RESI index variation grade gradually decreased, indicating that the overall performance of the quality of the eco-environment in the basin was gradually changing to a higher grade.

It can be seen from Figure 5 and Table 6 that the changes in the quality of the eco-environment in the Weihe River Basin were inconsistent in the different periods. From 2000 to 2007, the proportion of the area with eco-environment quality deterioration in the Weihe River Basin decreased significantly from 20.92% to 14.45%, and the proportion of the area without significant change increased to 64.77%, which demonstrates that the eco-environment quality in the Weihe River Basin significantly improved during this period. In Shaanxi and Gansu, the policy of returning farmland to forests and grassland was implemented in 2000, and the implementation of this project was more complete in 2007. Thus, the proportion of the area with significantly better eco-environment quality in Shaanxi and Gansu increased gradually during this period, while deterioration of the eco-environment occurred in Dingbian County and Wuqi County in the northern part of the river basin during this period. This was probably due to climatic factors and the fact that this area is on the Loess Plateau, which has more serious soil erosion and low vegetation coverage. After 2014, in terms of urban space development planning, the study area experienced rapid urbanization, forming an economic development center in the western region, and the construction land and road land gradually expanded, occupying the original grassland and arable land, and changing the original land use status. Affected by the urban development during this period, the soil conservation in the Weihe River Basin experienced a downward trend. However, compared with 2000, the overall eco-environment quality in the Weihe River Basin still improved. From 2017 to 2021, the areas with no significant change in the RSEI accounted for 85.14%.

### 3.3. Analysis of Driving Mechanisms of the Eco-Environment Quality in the Weihe River Basin

#### 3.3.1. Identification of Dominant Factors

In this study, four remote sensing ecological evaluation indicators (greenness indicator, wetness indicator, dryness indicator, and heat indicator) in 2021 were explored as internal factors, and 12 natural (elevation, Slope, PRE, and TEM), anthropogenic (GDP, POP, NLI, and HAI), and landscape (PD, SHDI, Split, and LSI) factors were explored as external drivers (Table 2) to determine the impact degrees and specific indicators of the eco-environment quality in the Weihe River Basin. A 1 km × 1 km grid was created within the study area, and the natural breakpoint method was used to classify the independent variables. Geographic probes were applied to explore the degree of influence of the individual factors and their interactions on the eco-environment quality.

(1) Factor detection analysis

Table 7 shows the factor detection results of the geographic detector. It can be seen that the explanation level of all the influencing factors on the eco-environment quality of the Weihe River Basin is significant at the 1% level, which indicates that all the 16 influence factors selected in this paper have a significant influence on the spatial differentiation of the eco-environment quality of the Weihe River basin. From the perspective of internal remote sensing factors, the greenness index dominates the regional eco-environment quality. From the perspective of external driving factors, the eco-environment quality of the Weihe River Basin is most affected by precipitation, and the q value of the precipitation factor is 0.428. Human activity intensity and nighttime light intensity can reflect regional social and economic development to a certain extent, and also have a very significant impact on the eco-environment quality, but the explanatory power of q is lower than that of remote sensing and topographic index. In addition, we found that the landscape pattern index also plays a crucial role in the eco-environment quality of the Weihe River Basin. Among them, the Shannon index can reflect the landscape richness of the region, and its q has the strongest explanatory power among the four landscape indexes.

(2) Interaction detection analysis

Table 8 shows the interaction detection results for each influencing factor. It can be seen that the influence of any two-factor interaction is greater than that of the single factors. The interaction detection results of all of the factors were two-factor enhancement, and there was no independent or weakening situation, indicating that the eco-environment in the Weihe River Basin was not the result of the action of a single factor but the result of interactions between the different factors, such as the natural factors, anthropogenic factors, and landscape pattern factors, and the remote sensing ecological evaluation indicators. The contributions of the interactions between the greenness index and the other factors were all greater than 0.812, which indicates that the influence of the factor with the greatest influence on the single-factor detection results will be significantly strengthened after the interaction detection with other factors. In summary, from the perspective of internal remote sensing indicators, the vegetation coverage in the Weihe River Basin and the regional climate had greater impacts on the quality of the eco-environment in the region, and the quality of the regional eco-environment was dependent on the natural factors. In terms of the external influences, precipitation and the quality of the eco-environment have an important association, and its interaction force with other indicators is greater than 0.428, while the intensity of human activities also has an important driving effect on the eco-environment quality, and its interaction force with each factor is also above 10%. Among them, the interaction between precipitation and HAI explained 55.8% of the spatial variation in RSEI, and the interaction between precipitation and elevation explained 57.1% of the spatial variation in RSEI. Overall, we found that climatic factors, topographic factors, and anthropogenic factors play a dominant role in the spatial distribution of regional RSEI variation.

#### 3.3.2. Spatial Variability of Drivers of RSEI Change

The results of the geodetection analysis (Table 6) showed that the values of all detection factors reached a significant 1%, indicating that the Weihe River basin RSEI is the result of the interaction of various elements in each type of driving force. In addition, considering the explanatory power of the collated values and combining them with the OLS regression results, we finally selected NDVI, which plays a dominant role in internal remote sensing ecological factors, and external influencing factors with different dimensions of influencing factors with q-values above 10% for further spatial heterogeneity analysis. These factors were specifically NDVI, Elevation, PRE, HAI, and SHDI.

An important prerequisite for conducting geographically weighted regression analysis is the existence of a strong spatial autocorrelation of the dependent variable, so this paper used ArcGIS 10.5 software to conduct a spatial Moran’s test for the RSEI in the Weihe River Basin from 2000 to 2021, and the results showed that all Moran’s I values for the RSEI from 2000 to 2021 were greater than 0, and all *p*-values were less than 0.001, indicating that there is a significant positive spatial autocorrelation of RSEI (Table 9). The MGWR model was used in this study to determine the spatial distribution of the effects of different drivers on RSEI changes. We took the 2021 Weihe River basin RSEI as an example and constructed OLS and GWR models using MGWR2.0, respectively. Combining the size of the study area and the sample size requirement of MGW2.0 software, a cell grid of 10,000 m × 10,000 m was created in this paper to explore the spatial relationship between each driver and the RSEI. Table 10 shows the performance comparison between the OLS and MGWR models. The R^2^ of the MGWR model is significantly higher than that of the OLS model, demonstrating the higher explanatory power and model fitness of the MGWR model. In addition, the lower AICc value of the MGWR model indicates that it possesses concise and more reliable regression estimates [49]. Therefore, MGWR can reflect the phenomenon more accurately compared to OLS.

**Figure 6:**
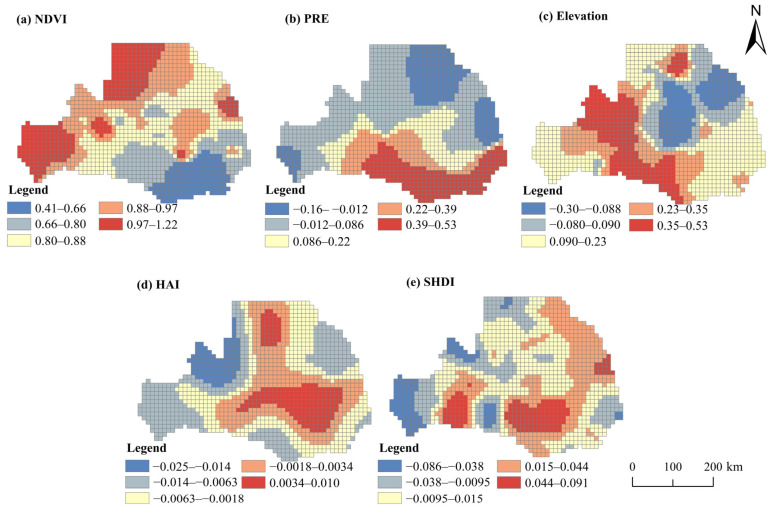
Spatial Heterogeneity of Influencing Factors of RSEI in Weihe River Basin.

The MGWR model was used to analyze the geospatial relationship between drivers and changes in RSEI. On the basis of the running results of MGWR2.0 software, we used the natural optimal breaking point classification method to visualize the regression coefficients of each influencing factor on the ArcGIS 10.5 platform (Figure 6). The correlation coefficient for each driver reflects the spatial response to RSEI changes. Among the internal remote sensing ecological factors, NDVI played a leading role in the change in RSEI; RSEI and NDVI were significantly positively correlated, and the correlation coefficient was higher in the northwest region of the Weihe River Basin. Among the natural factors, precipitation and elevation dominate the changes in RSEI, and the correlation coefficient of precipitation shows a decreasing trend from south to north. The southern part of the Weihe River basin is the northern part of the Qinling Mountains, which is rich in vegetation types, and the increase in precipitation creates good natural conditions for regional vegetation. The northern area is the Loess Plateau, with fragile ecological background and serious soil erosion. The increase in precipitation has less improvement on the eco-environment quality, and the extreme precipitation will also aggravate the current situation of soil erosion in the area, so the eco-environment management and vegetation restoration projects in the area should be continuously strengthened. However, the spatial relationship between RSEI and elevation was inconsistent, and the correlation coefficient was higher in the central part of the study area and lower in the peripheral areas, mainly because of the variable topography and significant spatial differences in natural elements in the study area. There was a significant negative correlation between RSEI and human factors, and the correlation coefficient decreased from the middle ring to the outside. In the landscape pattern index, the spatial impact of SHDI on RSEI varies greatly, and the overall performance is that the regional correlation coefficient of population agglomeration is positive, and gradually presents a negative correlation to the northwest region. There were obvious differences in the spatial changes in each driver and RSEI, which indicate that different factors dominate the changes in RSEI in different regions and zoning management is crucial to achieving sustainable management of RSEI.

## 4. Discussion

### 4.1. Exploring the Spatial and Temporal Evolution of the Eco-Environment Quality in the Weihe River Basin

Temporally, the quality of the eco-environment in the Weihe River Basin improved overall from 0.376 in 2001 to 0.414 in 2021. The medium and high RSEI areas increased significantly, by 26.42% and 27.70%, respectively. The Weihe River Basin was the first area on the Loess Plateau in which ecological management projects were implemented, and priority has been given to returning farmland to forests and grassland. Ecological restoration projects, such as reservoir construction and sediment interception, and some water diversion projects, such as “diverting red water to stone” and “diverting dry water to water,” have been implemented. The eco-environment quality has changed significantly, with the surface water area expanding and the vegetation coverage increasing significantly in about 62.3% of the area [70,71,72,73]. However, due to the fragile background of the eco-environment and low level of economic development, the ecological stability of the basin still needs to be improved, and a great deal of work is still required to achieve quality coordinated development of the basin’s eco-environment and socio-economic development.

Overall, the quality of the eco-environment in the Weihe River Basin has exhibited obvious synergistic effects with the NDVI and WET indicators, and their spatial changes were consistent. Before 2005, the project of returning farmland to forest was initially implemented, and the vegetation types started to change, but the ecological benefits of soil consolidation and water conservation were not obvious [74]. After 2010, the benefits of the reforestation and grassland restoration project began to emerge, some tree species matured, the spatial three-dimensional structure was highlighted, the watershed as a whole shifted into medium and high vegetation cover classes, the overall vegetation cover of the watershed increased, and the eco-environment quality improved significantly.

Spatially, the area that improved significantly during 2000–2021 was the northern bank of the Weihe River Basin and its two major tributaries, the Jing River and the Beiluo River. The eastern Ziwu Ridge is the watershed of the Jing and Beiluo rivers, and it contains dense secondary natural forests. The upper reaches of the Beiluo River Basin are loess hills and gullies, and the middle reaches are flanked by the Ziwu Ridge area and the Huanglong Mountain forest area. The quality of the environment in these areas improved more significantly after the implementation of a series of ecological projects, which demonstrates that the returning farmland to forest and grassland project had a significant effect on the vegetation restoration throughout the entire watershed.

In addition, the areas where the eco-environment quality deteriorated significantly during the study period were located in the Guanzhong Plain urban agglomeration, with Xi’an as the core, in the southern part of the Weihe River Basin. In terms of urban spatial development planning, the study area has undergone rapid urbanization and is becoming the center of economic development in the western region, with the concrete expansion of construction land and roads on the original grassland and arable land. This has affected the original land use conditions, and the soil retention in the Weihe River Basin has decreased. In 2012, the government put forward the strategy of vigorously promoting the construction of ecological civilization, and the study area has paid increasingly more attention to developing an ecological city. Green belts were added to the construction land, and the number of urban parks was increased, which helped offset the negative effects of urbanization on the regional eco-environment.

**Figure 7:**
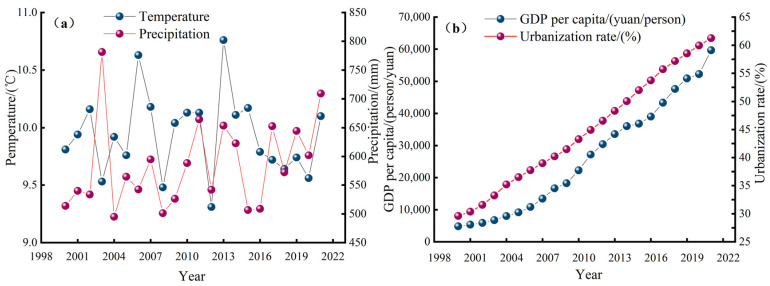
Annual per capita GDP, urbanization development change (**a**) and annual average climate change (**b**), in the Weihe River Basin.

In this study, it was found that the various factors affecting the eco-environment quality in the Weihe River Basin interacted, including the natural, economic, social, and landscape pattern factors. Due to climate change, the temperature and precipitation in the Weihe River Basin have changed greatly over the years (Figure 7a), which, to a certain extent, caused the fluctuations in the coupling of the regional water, air, and soil, thus affecting the quality of the regional eco-environment. In addition, in the Guanzhong Plain in the Weihe River Basin, urbanization led to the expansion of impervious surfaces, and the urban heat island effect became significant, which was reflected in the strengthening of the negative effect of the LST in this region and the decline in the quality of the eco-environment. In addition, the changes in vegetation coverage are closely related to human factors and natural factors. The eco-environment quality changes in the Weihe River region are more affected by natural factors in the northern regions with higher altitudes. During 2000–2021, the NDVI index in the region shifted to a high level, the high-altitude areas were less affected by human activities, and the eco-environment quality in the region improved significantly. Therefore, in order to ensure the sustainable development of national vegetation construction, the Weihe River Basin should be ecologically managed under rational and scientific planning, and in the future, research on the relationship between vegetation cover change and climate change should be further strengthened to help the development of green urbanization.

### 4.2. Analysis of Spatial Heterogeneity Factors Affecting RSEI

This paper can identify the dominant factors affecting RSEI and the interaction of each influencing factor through the function of geographic detector factor detection and interaction detection. Geodetection studies found that the eco-environment quality of the Weihe River basin is the result of the interaction of natural, economic and social, and landscape pattern factors. The synergistic effect of internal remote sensing greenness interacting with other factors is significantly enhanced, while the external influencing factors Elevation, PRE, HAI, and SHDI are the dominant external driving factors. The reason for this is that vegetation cover directly affects and responds to the regional environmental conditions, while the Weihe River basin is located in the inland northwest, with an arid climate, uneven precipitation variability, and fragile ecological background, and natural factors are the dominant drivers of the eco-environment quality in the region. There were clear spatial differences in the relationship between RSEI and the corresponding drivers. The global regression model cannot fully reflect the relationship between them. The GWR model can effectively overcome the spatial heterogeneity, but it can only reflect the optimal average scale of the spatially non-stationary relationship between the dependent variable and all independent variables. The MGWR model is an extension of GWR, which effectively solves the above problems by considering the optimal bandwidth of multiple independent variables to reflect the spatial non-stationary relationship [50,51]. We used the MGWR model to explore the relationship between RSEI and geospatial drivers.

As an important climatic factor, precipitation has the greatest influence among the external driving factors; anthropogenic factors have a significant negative impact on RSEI, and the vegetation factor is the main driving force for RSEI improvement. The adverse effects of human activities and climate change on ecosystem structure and processes are the main causes of ecosystem degradation [75,76]. The eco-environment quality in the northern part of the Weihe River Basin is relatively fragile and sensitive to human activities and climate change. Therefore, the negative correlation between RSEI and anthropogenic and climatic factors is stronger in the northern region (Figure 6). Analysis of the multi-year climate change in the Weihe River basin reveals a large multi-year variation in basin temperature and precipitation (Figure 7a), which to some extent exacerbates the soil erosion problem in the basin and thus has an important impact on the regional eco-environment quality, especially for the northern Loess Plateau region. In addition, in the Guanzhong Plain of the Weihe River Basin, the contradiction between man and land is prominent, and the population and economy are growing rapidly (Figure 7b). Therefore, the deterioration of eco-environment quality in this region is more affected by human factors.

Vegetation cover is the basis for the formation of ecosystem services in the basin. Vegetation restoration is conducive to improving the supply of regional ecosystem services, which is of great significance to the overall improvement of the quality of the regional eco-environment [74]. The Loess Plateau in the northern region has a fragile eco-environment, and large-scale vegetation restoration should be strengthened to reduce regional soil erosion. In the landscape pattern index, the spatial impact of SHDI on RSEI varies greatly, and the overall performance is that the regional correlation coefficient of population agglomeration is positive, and gradually presents a negative correlation to the northwest region. In areas with intensive human activities, a reasonable landscape pattern layout can further improve the regional eco-environment quality. Overall, the drivers selected in this study explained most of the variation in RSEI (Table 6 and Table 7), and the strength and nature of the correlation coefficients of each influencing factor varied significantly in terms of spatial variation (Figure 6). Therefore, for the Weihe River basin with a low regional economic level and fragile eco-environment quality, regional management should promote the sustainable development of national vegetation construction, improve the diversity of species in the basin, pay attention to the optimization of landscape pattern, and improve the resistance and resilience of the ecosystem. At the same time, future ecosystem management, planning, and decision making should focus on maintaining a balance between human activities and vegetation restoration.

### 4.3. Limitations and Future Directions

Using the GEE platform, based on MODIS image data, and supplemented by geographic detectors and MGWR in this study, the dominant factors affecting the quality of the regional eco-environment were comprehensively analyzed, and the spatial heterogeneity of each influencing factor was explored. The most prominent advantages of this remote sensing data-based eco-environment quality assessment method and the integrated multi-model driving mechanism exploration are the diversity of the data sources and the less subjective intervention, which overcomes the difficulty of obtaining long time series data for traditional eco-environment quality assessments in large-scale watersheds. At the same time, this paper provides a convenient method to capture the relationship between RSEI and driving factors from a geospatial perspective, and provides a reference for sustainable management of watershed eco-environment quality.

However, there were significant differences in the eco-environment quality changes within the Weihe River Basin, and a series of ecological projects have had large impacts on the regional eco-environment quality. The large-scale restoration of vegetation can affect the regional eco-environment through hydrological processes such as precipitation interception, soil infiltration, evapotranspiration, and climate change. Therefore, it is worthwhile to conduct a detailed investigation of the impact of the vegetation changes on the eco-environment in the Weihe River Basin in the future based on the intrinsic mechanisms of the regional climate and hydrological processes.

Due to the different ecological backgrounds in different study areas, there are significant spatial differences in the characteristics of regional natural resources. Land use change can reflect the differences in regional ecosystems to a certain extent. Therefore, in the construction of a future eco-environment quality evaluation model, the land use/cover change data can be combined, and ecological factors can be selected according to the natural characteristics of different land-use types. In order to more accurately evaluate the quality of the regional eco-environment.

## 5. Conclusions

Based on the GEE platform, this study monitored and analyzed the eco-environment quality of the Weihe River Basin from 2001 to 2021, and used geographic detectors and multiple linear regression models to explore the influencing factors of the eco-environment quality of the Weihe River Basin. Based on the GEE platform, this study monitored and analyzed the eco-environment quality of the Weihe River basin from 2001 to 2021 and analyzed the drivers of eco-environment quality from a geospatial perspective in conjunction with a geographic probe and MGWR model. The results show that the overall RSEI of the Weihe River Basin exhibited a fluctuating upward trend from 2000 to 2021. Spatially, the quality of the eco-environment in the Weihe River Basin was high in the south and low in the north. In terms of trends and levels of change, the overall eco-environment quality in the Weihe River Basin improved during the study period and can be divided into two periods of rapid improvement (2000–2014) and slow turnaround (2014–2021). The eco-environment quality in the northern part of the study area improved significantly, and ecological engineering and policy control in this region should be strengthened. However, in the southern part of the Weihe River Basin where urbanization was relatively developed, the eco-environment quality deteriorated, and all of the stakeholder groups should pay more attention to eco-environment protection and high-quality development in this region. The natural factors, human factors, and landscape factors jointly controlled the changes in the eco-environment in the basin. All of the interactions between the influencing factors had a stronger influence than those of the individual factors. There were significant differences between the individual drivers and the spatial variation in RSEI, suggesting that different factors dominate the variation in RSEI in different regions, and zonal management is crucial to achieving sustainable management of RSEI.

In general, on the basis of the fragile ecological background of RSEI in the Weihe River Basin, human factors and vegetation factors are the main driving factors of regional ecosystem changes, which means that optimizing human activities and increasing vegetation will best improve the quality of the regional eco-environment. At the same time, in areas with intensive human activities, rational allocation of landscape elements will play an important role in promoting the improvement of regional eco-environment quality.

## Figures and Tables

**Table 1:** The data source and index descriptions of four ecological components.

Indicator	Remote Sensing Product	Spatial Resolution	Temporal Resolution	Description of Indicator
NDVI	MODIS09A1	500 m	8 d	MODIS09A1 images provide surface spectral albedo estimates in Terra MODIS bands 1–7 corrected for atmospheric conditions such as gases, aerosols, and Rayleigh scattering
WET				
NDSI				
LST	MODIS11A2	1000 m	8 d	MODIS products remove cloud-contaminated pixels from Level 2 and Level 3 surface temperature products

**Table 2:** Selection of driving factors and data processing.

	Driving Factors	Sources	Processing
Natural factors	Elevation	https://search.asf.alaska.edu/ (accessed on 18 April 2022)	digital elevation model (DEM) Advanced Land Observing Satellite (ALOS) 12.5 m × 12.5 m data from the National Aeronautics and Space Administration (NASA) EARTHDATA website
	Slope	https://search.asf.alaska.edu/ (accessed on 18 April 2022)	Calculated based on DEM data
	Temperature (PRE)	http://data.cma.cn/ (accessed on 25 April 2022)	Kriging interpolation is performed on the meteorological station data to obtain raster data with a resolution of 1000 m × 1000 m
	Precipitation (TEM)	http://data.cma.cn/ (accessed on 25 April 2022)	
Landscape factors	Patch Density (PD)	Calculation based on LULC data, in the FRAGSTATS software platform, the calculation is carried out by the moving window method [60,61]	PD = $\sum_{i=1}^{n}$n_i_A where n is the total number of plaques, A is the total area of plaques, and i is the type of plaques; PD is the number of plaques of a certain type per unit area, which can reflect the density of plaques.
	Shannon’s Diversity Index (SHDI)		SHDI = −$\sum_{i=1}^{m}$P_i_lnP_i_ where m is the total number of landscape types, P_i_ is the proportion of the area of landscape type i; SHDI is a measurement index based on information theory, which can reflect the diversity of landscapes.
	Splitting Index (Split)		Split = D_i_/A_i_ where D_i_ is the distance index of landscape type i, and A_i_ is the area index of landscape type i; landscape separation degree refers to the degree of separation of individual distributions of different patches in a certain landscape type.
	Landscape Shape Index (LSI)		LSI = 0.25E/A^1/2^ where E is the total length of all patch boundaries in the landscape and A is the total area of the landscape; LSI refers to the degree of deviation between the shape of a patch in the study area and a square of equal area, the higher the value, the more complex the shape of the patch in the landscape.
Anthropogenic Factors	Human activity intensity (HAI)	Xu et al., 2016 [62]	HAI = $\sum_{i=1}^{n}$S_i_C_i_/S where S_i_ is the area of the ith land use type, C_i_ is the human activity intensity coefficient of the ith land use type, n is the number of land use types, and S is the total area of the region.
	Nighttime light intensity (NLI)	(https://eogdata.mines.edu/nighttime_light/) (accessed on accessed on 20 April 2022)	ArcGIS Spatial analysis function
	Gross domestic product (GDP)	China County Statistical Yearbook	ArcGIS Spatial analysis function
	Population density (POP)	China County Statistical Yearbook	ArcGIS Spatial analysis function
Other data	Per capita gross domestic product (RMB/person)	China Urban Statistical Yearbook (http://www.stats.gov.cn/) (accessed on accessed on 20 April 2022)	The ratio of total GDP to average annual population
	Urbanization rate (%)	China Urban Statistical Yearbook (http://www.stats.gov.cn/) (accessed on accessed on 20 April 2022)	The proportion of urban population to total population
	Annual average temperature	http://data.cma.cn/ (accessed on accessed on 23 April 2022)	ArcGIS Spatial analysis function
	Annual average precipitation	http://data.cma.cn/ (accessed on accessed on 23 April 2022)	ArcGIS Spatial analysis function

**Table 3:** Results of principal component analysis for each indicator in the Weihe River Basin for the RSEI during 2000–2021.

Year	Indicators	PC1	PC2	PC3	PC4
2000	NDVI	0.671	0.262	−0.557	0.414
	WET	0.461	0.477	0.259	−0.702
	NDSI	−0.064	−0.411	−0.701	−0.580
	LST	−0.577	0.732	−0.363	−0.016
	Eigenvalues	0.055	0.009	0.004	0.002
	Eigenvalue contribution rate	78.93%	12.55%	5.63%	2.89%
2003	NDVI	0.699	0.235	−0.404	−0.541
	WET	0.474	0.490	0.146	0.717
	NDSI	−0.058	−0.311	−0.849	0.424
	LST	−0.532	0.780	−0.309	−0.118
	Eigenvalues	0.044	0.015	0.006	0.003
	Eigenvalue contribution rate	65.20%	21.88%	8.66%	4.26%
2007	NDVI	0.693	0.157	−0.431	0.556
	WET	0.459	0.317	−0.190	−0.808
	NDSI	−0.213	−0.580	−0.769	−0.167
	LST	−0.514	0.734	−0.433	0.098
	Eigenvalues	0.063	0.012	0.006	0.002
	Eigenvalue contribution rate	75.30%	14.87%	7.19%	2.64%
2010	NDVI	0.708	0.056	−0.391	0.586
	WET	0.505	0.225	−0.246	−0.796
	NDSI	−0.156	−0.776	−0.596	−0.135
	LST	−0.468	0.586	−0.657	0.072
	Eigenvalues	0.047	0.017	0.008	0.003
	Eigenvalue contribution rate	63.14%	23.32%	10.19%	3.35%
2014	NDVI	0.754	0.276	0.502	−0.321
	WET	0.423	0.345	−0.333	0.769
	NDSI	−0.020	−0.529	0.660	0.533
	LST	−0.502	0.725	0.448	0.145
	Eigenvalues	0.047	0.014	0.004	0.002
	Eigenvalue contribution rate	0.71	0.21	0.05	0.03
2017	NDVI	0.686	0.287	0.450	0.495
	WET	0.462	0.349	−0.030	−0.815
	NDSI	−0.055	−0.560	0.771	−0.299
	LST	−0.560	0.695	0.450	−0.036
	Eigenvalues	0.051	0.016	0.003	0.002
	Eigenvalue contribution rate	70.77%	21.96%	4.48%	2.80%
2021	NDVI	0.642	0.350	0.468	0.496
	WET	0.413	0.440	−0.051	−0.796
	NDSI	−0.063	−0.474	0.807	−0.346
	LST	−0.643	0.678	0.356	0.018
	Eigenvalues	0.057	0.012	0.005	0.002
	Eigenvalue contribution rate	73.99%	16.08%	7.06%	2.88%

**Table 4:** Average correlation of each index.

Year	RESI	NDVI	WET	NDSI	LST
2000	0.75	0.60	0.64	0.25	0.50
2003	0.69	0.51	0.51	0.20	0.34
2007	0.81	0.69	0.69	0.43	0.52
2010	0.73	0.56	0.58	0.22	0.37
2014	0.68	0.53	0.57	0.25	0.43
2017	0.70	0.59	0.61	0.29	0.45
2021	0.73	0.58	0.62	0.24	0.46
Average	0.73	0.58	0.60	0.27	0.44

**Table 5:** Statistics of the areas (10^3^ km^2^) and proportions (%) of the RSEI classification levels in the Weihe River Basin during 2001–2021.

	2000		2003		2007		2010		2014		2017		2021	
RSEI Grading	Area	Pro Portion	Area	Pro Portion	Area	Pro Portion	Area	Pro Portion	Area	Pro Portion	Area	Pro Portion	Area	Pro Portion
Extremely low RSEI	21.75	16.25	12.06	9.01	19.72	14.73	5.28	3.94	1.40	1.04	8.53	6.37	8.89	6.64
Low RSEI	60.66	45.31	80.02	59.77	58.45	43.65	60.67	45.32	48.00	35.85	59.06	44.11	62.36	46.58
Medium RSEI	34.29	25.61	26.55	19.83	37.92	28.32	48.94	36.55	55.07	41.13	42.69	31.89	43.35	32.38
High RSEI	14.19	10.60	13.88	10.37	16.21	12.11	17.77	13.27	25.30	18.89	20.97	15.66	18.12	13.53
Extremely low RSEI	2.99	2.24	1.38	1.03	1.59	1.19	1.22	0.91	4.13	3.08	2.63	1.97	1.17	0.88
Total	133.89	100	133.89	100	133.89	100	133.89	100	133.89	100	133.89	100	133.89	100

**Table 6:** Changes in the areas (10^3^ km^2^) and percentages (%) of the RSEI classes in the Weihe River Basin from 2000 to 2021.

	2000–2003		2003–2007		2007–2010		2010–2014		2014–2017		2017–2021	
RSEI Change	Area	Proportion	Area	Proportion	Area	Proportion	Area	Proportion	Area	Proportion	Area	Proportion
very significant deterioration (˂−0.2)	0.04	0.03	0.00	0.00	0.01	0.00	0.03	0.02	0.00	0.00	0.01	0.00
Severely deteriorated (−2.0 to −1.5)	0.71	0.53	0.12	0.09	0.01	0.01	0.13	0.09	0.47	0.35	0.03	0.02
Moderate variation (−1.5 to −1.0)	5.27	3.94	3.28	2.45	0.15	0.11	0.74	0.55	11.79	8.81	0.55	0.41
Slight deterioration (−1.0 to −0.5)	21.98	16.42	15.95	11.91	3.10	2.32	3.79	2.83	53.03	39.60	14.56	10.88
No significant changes (−0.5 to 0.5)	78.21	58.42	86.72	64.77	80.61	60.21	50.40	37.65	67.52	50.43	114.26	85.34
Slightly improved (0.5~1.0)	22.47	16.78	21.56	16.10	34.77	25.97	64.37	48.07	1.06	0.79	4.40	3.28
Moderately better (1.0~1.5)	4.96	3.70	5.00	3.73	13.94	10.41	14.13	10.55	0.02	0.02	0.08	0.06
obvious change (1.5~2.0)	0.25	0.19	1.06	0.79	1.30	0.97	0.31	0.23	0.00	0.00	0.00	0.00
improved significantly (>2.0)	0.00	0.00	0.20	0.15	0.01	0.00	0.00	0.00	0.00	0.00	0.00	0.00
Total	133.89	100.00	133.89	100.00	133.89	100.00	133.89	100.00	133.89	100.00	133.89	100.00

**Table 7:** Analysis of the results of the influencing factor detection.

	Remote Sensing Index				Natural Factors			
	WET	NDVI	NDSI	LST	Slpoe	Elevation	Pre	Tem
q statistic	0.676 ***	0.812 ***	0.061 ***	0.787 ***	0.050 ***	0.103 ***	0.428 ***	0.066 ***
	**Anthropogenic Factors**				**Landscape Factors**			
	POP	GDP	HAI	NLI	PD	SHDI	SPLIT	LSI
q statistic	0.012 ***	0.009 ***	0.111 ***	0.018 ***	0.014 ***	0.101 ***	0.015 ***	0.003 ***

Note: *** means that the explanatory power of each factor to RSEI is significant at the 1% level.

**Table 8:** Interaction detection results for each influencing factor.

	WET	NDVI	NDSI	LST	Slpoe	Elevation	Pre	Tem	POP	GDP	HAI	NLI	PD	SHDI	Split	LSI
WET	0.677															
NDVI	0.849	0.812														
NDSI	0.729	0.843	0.061													
LST	0.932	0.946	0.849	0.787												
Slpoe	0.788	0.839	0.219	0.817	0.066											
Elevation	0.848	0.881	0.293	0.878	0.141	0.103										
Pre	0.716	0.843	0.467	0.88	0.541	0.571	0.428									
Tem	0.849	0.889	0.278	0.879	0.125	0.168	0.625	0.093								
POP	0.731	0.816	0.089	0.79	0.081	0.108	0.453	0.097	0.012							
GDP	0.744	0.821	0.1	0.809	0.069	0.129	0.463	0.119	0.017	0.009						
HAI	0.765	0.83	0.26	0.81	0.139	0.185	0.558	0.178	0.116	0.124	0.111					
NLI	0.736	0.815	0.091	0.788	0.072	0.11	0.46	0.1	0.021	0.019	0.12	0.018				
PD	0.687	0.819	0.085	0.795	0.084	0.124	0.443	0.113	0.03	0.025	0.129	0.035	0.015			
SHDI	0.69	0.82	0.195	0.8	0.194	0.135	0.447	0.127	0.137	0.132	0.138	0.142	0.122	0.101		
Split	0.689	0.82	0.088	0.797	0.085	0.126	0.445	0.117	0.03	0.026	0.133	0.035	0.015	0.122	0.015	
LSI	0.687	0.819	0.078	0.797	0.071	0.116	0.442	0.104	0.016	0.013	0.125	0.022	0.018	0.124	0.018	0.003

**Table 9:** Global Moran Test of RSEI in the Weihe River Basin.

	2000	2003	2007	2010	2014	2017	2021
Moran’I	0.871	0.841	0.880	0.865	0.858	0.867	0.862
Z	46.090	44.507	46.546	45.754	45.376	45.877	45.618
*p*	0	0	0	0	0	0	0

**Table 10:** Comparison of estimation results between OLS (Ordinary Least Squares) and MGWR (Multiscale Geographically Weighted Regression) model.

OLS Model					MGWR Model				
	Coefficient	*t* Value	*p* Value	VIF	Mean	Std	Min	Med	Max
NDVI	0.887	93.394	0.000	2.497	0.862	0.141	0.410	0.878	1.219
PRE	0.089	9.291	0.000	2.519	0.136	0.187	−0.160	0.066	0.535
Elevation	0.279	43.227	0.000	1.152	0.175	0.192	−0.298	0.192	0.531
HAI	0.039	6.453	0.000	1.015	−0.004	0.007	−0.025	−0.004	0.010
SHDI	0.011	1.676	0.094	1.159	0.007	0.032	−0.086	0.006	0.091
R^2^	0.949				0.993				
Adj. R^2^	0.949				0.992				
AICc	−189.425				−2434.734				

Note: VIF is the coefficient of variance expansion. If all VIFs are less than 10, it means that the model has no multicollinearity problem; min, max, std, and med represent the minimum, maximum, standard deviation, and median of the estimated coefficients of the GWR model, respectively.

## Data Availability

The MODIS data can be downloaded from the GEE (https://developers.google.com/earth-engine/datasets/catalog/modis accessed on 15 April 2022).
